# FASTER EPIGENETIC AGING AND INCREASED RISK OF PROSPECTIVE MORBIDITY AND MORTALITY FOR U.S. VETERANS

**DOI:** 10.1093/geroni/igae098.4293

**Published:** 2024-12-31

**Authors:** Kyle Bourassa

**Affiliations:** Durham VA Medical Center, Durham, North Carolina, United States

## Abstract

Epigenetic measures of aging have promise as biomarkers of future morbidity and mortality, but require validation in real-world medical settings. To provide evidence of this validation, we investigated whether an epigenetic measure of aging—DunedinPACE—was associated with prospective health for 2,216 post-9/11 U.S. military veterans enrolled in the Post-Deployment Mental Health Study (472 women, 1,744 men; 1,077 non-Hispanic Black veterans, 1,139 non-Hispanic White veterans). Prospective health outcomes included chronic disease burden (Charlson comorbidity index), predicted annual VA healthcare costs (Nosos scores), chronic disease incidence, and mortality—all assessed using VA electronic health records (EHR). Veterans averaged 37.4 years old at enrollment and with an average of 13.1 years of EHR follow-up. Veterans with faster DunedinPACE aging scores developed greater chronic disease burden and greater predicted healthcare costs over the subsequent 5, 10 year, and 15 years. Faster aging scores were associated with increased risk for incident myocardial infarction, stroke, diabetes, cancer, liver disease, and renal disease, as well as greater risk of mortality due to all-causes and death due to chronic disease. Results remained when controlling for demographic and technical covariates, as well as self-reported smoking and smoking methylations scores, replicated when stratifying by sex and race/ethnicity, and replicated when using an alternative measure of epigenetic aging (PC-GrimAge). These findings suggest DunedinPACE is a biomarker of accelerated aging that associated with prospective morbidity and mortality across multiple chronic diseases and organ systems. With further refinement, epigenetic aging scores have the potential to be useful clinical biomarkers of future health.

